# Understanding burnout

**Published:** 2009

**Authors:** R. Srinivasa Murthy

**Affiliations:** Professor of Psychiatry (Retd), Bangalore, India. E-mail: murthy_srinivasar@yahoo.co.in

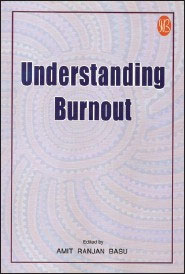



During the last half century, stress has come to dominate among the factors that are related to matters of health and disease. Understanding of stress can be compared with the earlier breakthroughs in medicine with the discovery of microbes about three centuries back and the discovery of biochemical substances (vitamins, hormones, etc.) during the last century. The focus on stress has been a reflection of the larger changes that have occurred in the society, like industrialisation, urbanisation, growth in information technology and communications. This shift in focus from microbes to ‘stress’ is referred to in a recent book as follows:

*‘Is stress a deadly disease on the rise in modern society? Are good friends the best medicine? Can mind–body practices from the East help us become well? Many of us think so. Doctors and drugs are often not enough, we think, we must also seek the cure within’*.[1]

Along with the science of stress came the precognition of the phenomenon of ‘burnout.’ Burnout is a complex psych–physiological syndrome represented by feelings of anxiety, tension, mental fatigue, physical exhaustion and a loss of concern for the people with whom one is living and working. It emerges as a result of chronic stress. Burnout is used with reference to a decrease in the physiological capabilities of athletes, coaches and managers to deal with stressful situations.

The book UNDERSTANDING BURNOUT focuses on the differences between the stress discourse and burnout from various contexts.

The book is divided into two sections. The first section, titled perspectives, covers the scope of the subject in five chapters. Beginning with a chapter on the contemporary views of burnout, the other chapter's present burnout and employee retention, work-life balance on employee commitment and the Indian model of executive burnout.

The second section has case studies from different sectors and different parts of the world. The coverage includes burnout in the medical profession, with examples of burnout from Hongkong, Iran, Brazil, USA and the Netherlands.

A salient observation from these chapters is that burnout is the result of overwork, repetitive work and monotony in work. In some countries, there are special terms referring to overwork exhaustion, like karoshi in Japanese. Work-life balance can be helpful both to the employees and to the employers. Organisations have an important role in shaping the behavior of individuals. In India, it is the high-achieving executives who suffered from high degrees of burnout. Consequences of burnout range from relationship problems to substance abuse and suicide. Hongkong, among the countries of Asia, has the highest incidence of staff burnout.

In Iran, the staff burnout was related to role overload, role expectation conflict, inter-role distance, resource inadequacy, role stagnation and role isolation. Among medical professionals, oncologists have very high rates of burnout. Minority students in the USA reported a lower sense of personal accomplishment and quality of life.

The overview chapter presents a very detailed historical account of the development of the concept and the different forces that have shaped to define the boundaries. The evolutionary nature of the concept is well documented.

In India, there is recognition of the role of the organisation in assisting staff to achieve a work-life balance. In a recent review of this area from India, it is recorded that there are specific programmes of work-life balance in Texas Instruments, Philips software centre, Eli Lilly (India), NIIT, IBM (India), Price Waterhouse Coopers in India, Infosys, ICICI, Procter and Gamble and BPO Industry. The measures have ranged from work options to leave options, child care facilities, video conferencing to reduce travel, not scheduling work events on school holidays and so on.[2]

It is also to be noted that in the last decade there is an explosion of alternative stress management initiatives (e.g., Transcendental Meditation, Art of Living, Vipasana etc.) by a wide variety of professionals and people. The wide use and popularity speaks of a need, if nothing else.

Dr. Amit Ranjan Basu, who has edited the book, has done a valuable service to the professions of mental health and management. The book presents opportunities for propagation of mental health principles in the community. The model used in the burnout is one of ‘normalcy’ of the experience rather than the ‘deviancy’ concept. Psychiatrists will do well to understand developments in this field and work toward integrating these interventions, in addition to their clinical care activities.

The book would be valuable for libraries of Departments of Psychiatry, Clinical Psychology and Social Work and institutions working with human resources development.

## References

[1] Harrington A (2008). The cure within-a history of mind-body medicine.

[2] Mohanty M (2008). Work-life balance: Issues and experiences. Focus Int J Manage Digest.

